# Symbiotic 8: Beyond Symbolic Execution (Competition Contribution)

**DOI:** 10.1007/978-3-030-72013-1_31

**Published:** 2021-02-26

**Authors:** Marek Chalupa, Tomáš Jašek, Jakub Novák, Anna Řechtáčková, Veronika Šoková, Jan Strejček

**Affiliations:** 8 Eindhoven University of Technology, Eindhoven, The Netherlands; 9 Aalborg University, Aalborg East, Denmark; 10 Masaryk University, Brno, Czech Republic; 11 Brno University of Technology, FIT, Brno, Czech Republic

## Abstract

Symbiotic  8 extends the traditional combination of static analyses, instrumentation, program slicing, and symbolic execution with one substantial novelty, namely a technique mixing symbolic execution with k-induction. This technique can prove the correctness of programs with possibly unbounded loops, which cannot be done by classic symbolic execution. Symbiotic  8 delivers also several other improvements. In particular, we have modified our fork of the symbolic executor Klee to support the comparison of symbolic pointers. Further, we have tuned the shape analysis tool Predator (integrated already in Symbiotic  7) to perform better on llvm bitcode. We have also developed a light-weight analysis of relations between variables that can prove the absence of out-of-bound accesses to arrays.

## 1 Verification Approach

Symbiotic is a program analysis framework that combines fast static analyses with code instrumentation and program slicing to speed up the code verification which is then performed by symbolic executor Klee [3] (or, alternatively, by another supported verification tool). The main improvement in Symbiotic 8 is a new verification technique combining symbolic execution with k-induction [8] that we call *KindSE*.

**Symbolic execution with k-induction (KindSE)** *KindSE* applies the idea of k-induction [8] to paths of the control flow graph. The approach can be roughly described by the following three steps.

1. Set $k$ to 1. Let $P$ be the set of all paths in the control flow graph of length $k$ that end in an error location.
2. Use symbolic execution to execute every path $\pi \in P$. If the symbolic execution says that $\pi$ is infeasible, remove $\pi$ from $P$. If $\pi$ is feasible and it starts in the initial location, report that the program is incorrect.

* This work has been supported by the Czech Science Foundation grant GA20-07487S.
✉ Jury member and the corresponding author: `chalupa@fi.muni.cz`.

3. If $P$ is empty, the control flow graph contains no feasible path of length $k$ (or more) leading to an error location and thus we report that the program is correct. If $P$ is not empty, we replace each path $\pi \in P$ by paths of length $k+1$ that have $\pi$ as its suffix, increase $k$ by one, and go to step 2.

To improve the performance, we further extended the algorithm to summarize loop iterations. If we process a program location that is a loop header, we start unwinding the loop backwards. We over-approximate the states that we get in every loop iteration to cover more than one iteration if possible. If we are successful, the summarized loop states form an inductive invariant, which can help to prove that no error location is reachable from the loop header in $k$ steps. Our loop summarization does not handle nested loops (in this case we fall-back to the algorithm without loop summarization) and calls of functions. To fix the latter restriction, we inline all procedures (if possible) before running KindSE.

KindSE is implemented in our prototype tool Slowbeast [1] which we integrated into Symbiotic 8. The tool now supports only the `unreach-call` property. Slowbeast can also work as a standard symbolic executor (without k-induction), but it is noticeably slower than Klee and it has some limitations. However, it supports symbolic floating point arithmetics, which Klee does not.

**Workflow of Symbiotic 8** As the first step, a given program is translated to llvm [6]. If the program contains a call to `pthread_create`, Symbiotic returns `unknown` as it cannot handle parallel programs. The rest of the workflow then depends on the verified property, as indicated in Figure 1.

For `unreach-call` property, we call slicer to remove instructions that have no influence on the property and run Klee. If Klee does not decide in 222 seconds, we run KindSE in Slowbeast. If it fails, we run Klee again and if it also fails, we run Slowbeast as a standard symbolic executor. If some tool says

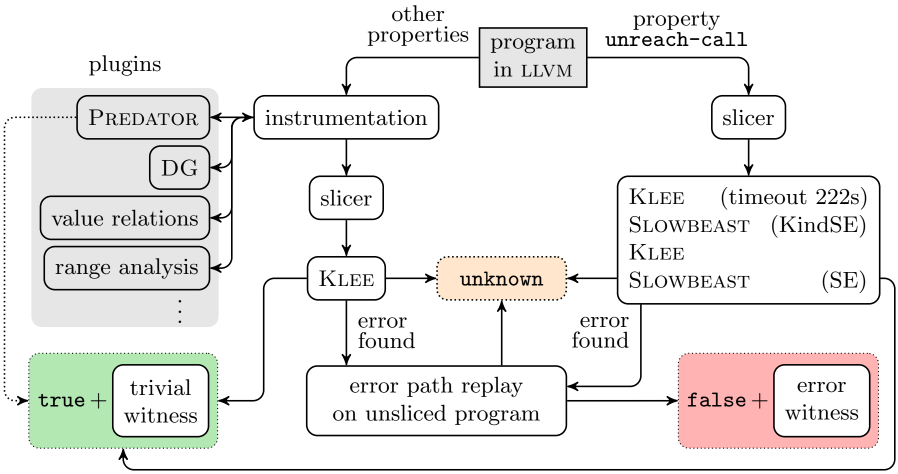

**Fig. 1.** The workflow of Symbiotic 8

that the specified call is unreachable, we return `true` with the trivial witness. If we detect that the specified call is reachable, we try replaying the error path on the unsliced program. If the replay confirms that the call is reachable, we return `false` with the error witness generated from the replay.

For other properties, we instrument the program with the help of various analyses. For example, when checking memory safety, we use Predator [5], DG [4], and a values-relations analysis to detect potentially unsafe instructions. If Predator says that all instructions are safe, we directly return `true`. Otherwise, we slice the program with respect to potentially unsafe instructions and call Klee. The rest of the process is identical to the previous case.

## 2 Software Architecture

All components of Symbiotic 8 use llvm 10 [6]. Scripts that call and control the components according to a given configuration are written in Python.

Instrumentation module is written in C++. In Symbiotic 8, we have newly integrated a values-relations analysis as a plugin into instrumentation. This analysis is able to prove valid some accesses into arrays. We have also improved llvm frontend of Predator [5] to perform similarly well as the gcc frontend.

Program slicing module is written in C++ and is build around the library DG [4]. This year, we sped up the slicer by using more efficient data structures in pointer analysis and by using function summaries in data dependence analysis.

We use our own fork of Klee [3] that differs from the upstream Klee mainly in using segment-offset pointer representation which allows for better handling of symbolic pointers and symbolic-sized allocations. This year, we mended handling of symbolic pointers and added support for comparison of symbolic addresses.

Tool Slowbeast [1] is written in Python. Both, Klee and Slowbeast use Z3 [7] as the SMT solver.

## 3 Strengths and Weaknesses

Symbolic execution may be very efficient in finding bugs but suffers from the *path explosion problem* which may prevent it from fully analyzing programs with high level of branching. We alleviate this problem by using program slicing. However, in the presence of unbounded loops or infinite execution paths, program slicing does not help unless it removes the unbounded computation from the program. Indeed, classical symbolic execution is unable to verify such programs at all.

To fight the inability of symbolic execution to verify unbounded programs, we use *KindSE*. However, its implementation in Slowbeast is still not fully matured and it handles only a very restricted set of programs.

**Results of Symbiotic 8 in SV-COMP 2021** Symbiotic 8 won *MemSafety* and *SoftwareSystems* categories [2]. In the *MemSafety* category, we lost many points in the new *MemSafety-Juliet* subcategory. These benchmarks contain threads and Symbiotic immediately answered *unknown* due to the syntactic check mentioned in Section 1. However, most of these benchmarks actually do not spawn any thread and thus Symbiotic could analyze them. The victory in *SoftwareSystems* category is mainly due to the dominance on the new *uthash* benchmarks.

This year, over 500 correct answers produced by Symbiotic were not confirmed. Some of these cases must be accounted to the fact that Symbiotic generates only trivial correctness witnesses. However, there are also unconfirmed answers because of missing witnesses, which turned out to be a bug in Slowbeast integration. Unfortunately, these include all 99 benchmarks that were newly proved correct by KindSE, from which 85 were in the *ReachSafety-Loops* subcategory. We had also many unconfirmed witnesses for non-termination violation that still need to be investigated.

Symbiotic had 16 incorrect answers: 14 incorrect *true* in *Termination* category and 2 incorrect *false* in *ReachSafety-Floats*. All of them were caused by last-minute commits that were fixed shortly after the submission deadline. Because of these mistakes, Symbiotic ended up on the 4th place instead of on the 2nd in the *Termination* category.

In the *Overall* meta-category, Symbiotic traditionally took the 4th place as every year since 2018.

## 4 Tool Setup and Project Contributors

The archive is available at https://doi.org/10.5281/zenodo.4483882. Run Symbiotic as:

```
bin/symbiotic --sv-comp --prp <prpfile> [--32] <source>
```

The option `--prp` sets the verified property and `--32` tells Symbiotic to assume 32-bit architecture (64-bit architecture is assumed by default).

## 5 Software Project and Contributors

Symbiotic 8 for SV-COMP 2021 has been developed by Marek Chalupa, Tomáš Jašek, Jan Novák, and Anna Řechtáčková under the supervision of Jan Strejček. Veronika Šoková provided a valuable help with adjusting Predator modifications. Symbiotic is available under the MIT license. All the external components that the tool uses are also available under open-source licenses that comply with SV-COMP's policy for the reproduction of results. The source code of Symbiotic can be found at:

https://github.com/staticafi/symbiotic
